# A Case of Intestinal Intussusception

**Published:** 1886-09

**Authors:** Christopher Elliott

**Affiliations:** Physician to the Bristol Hospital for Sick Children and Women


### A CASE OF INTESTINAL INTUSSUSCEPTION.

By CHRISTOPHER ELLIOTT, M.D., Physician to the Bristol Hospital for Sick Children and Women.

On December 4th, 1885, I was asked by a barber to visit his son, aged 8 years, who had been taken ill suddenly that morning about five o'clock with sharp abdominal pain of a paroxysmal character, and followed by sickness. When I arrived at the house I found the boy was somewhat easier, and in less pain. Temperature 98, pulse 76.

The abdomen was resonant upon percussion, and neither palpation nor deep pressure with the hand upon it caused any increase of pain. Learning that the boy had been indulging in sweets and apples the day before, I looked upon the case as one of ordinary colic, and ordered a dose of castor oil, and the patient to be kept on a milk diet.

In the evening I was summoned again hurriedly, and found my patient in great pain. The castor oil had been given, but was almost immediately vomited. There had been several attempts at defæcation, but only a large quantity of bloody mucus had been passed. Temperature 98·8°, pulse 80. I ordered hot fomentations to the abdomen, and prescribed small doses of belladonna, alternately with pulv. ipecac. co., every two hours.

Next morning the patient's condition was much the same. He had had a restless night, vomiting each dose of the belladonna mixture, although he retained the Dover powders. There had been much tenesmus and a further passage of bloody mucus from the rectum. Temperature 98·8°. The lower half of the abdomen was dull, and the dulness extended somewhat higher up on the left side. Believing I had to deal with a case of intestinal intussusception, I asked for and obtained the consent of the boy's father for his admission into the Hospital for Children and Women. There in the afternoon I had him placed under the influence of chloroform, and was able to satisfy myself as to the presence of a "sausage-like" tumour, of 2—3 inches in length and the thickness of the fore-finger, in the left iliac region. The tumour could not, however, be felt with the finger in the rectum. I now tried to reduce the invagination by the insufflation of air up the rectum by means of an ordinary kitchen bellows, to which I attached a short piece of rubber tubing, but without success. I next gave an enema of warm water, having the patient held in an inverted position, but this also failed to reduce the tumour. I then resumed the insufflation of air, but after steadily persevering with it for fully fifteen minutes, I was afraid to do more then, and determined to obtain the advice of my colleagues.

The same evening we met in consultation, when it was decided to give another enema, and failing that, abdominal section should be performed. Fortunately for the patient the latter proceeding was not necessary, as, at Mr. Norton's suggestion, cold water was employed; and its use was followed with such evident success, that after over a quart of water had been injected the tumour was found to have disappeared, and the intestines could be plainly seen distended under the parietes. The patient was put to bed and given small and frequent doses of pulv. ipecac. co. No food was allowed by the mouth, but enemata of beef tea and milk and brandy were given instead.

Next morning (December 6th) the temperature was 98°, pulse 72, and the patient had slept tolerably well. The abdomen was very tender, but the bowels had acted naturally, the motion being liquid and containing small pieces of hard solid fæces, but free from blood. As the tongue was very furred and the patient complained of thirst, the Dover powders were stopped, and belladonna was given instead.

On December 8th the bowels acted again of themselves, and a large solid motion was passed without pain.

On the 12th the patient was free from all pain and tenderness.

On the 14th he was allowed to get up.

On the 16th he was discharged well.

Such, gentlemen, are the notes of the case, which I

have thought to be of sufficient interest to read before you this evening,* and I have brought them forward also in the hope of eliciting from others their experiences. According to Mr. Durham (*vide* Quain's *Dictionary of Medicine*), one-fourth of the cases of intestinal intussusception on record have occurred in the first year, and more than one-half during the first seven years of life, so that my patient was slightly above the usual age. Without a post-mortem examination it is difficult to say exactly what part of the intestinal tract was involved, but most probably the ileum and cæcum passed into the colon, as the presence of the "sausage-like tumour" is considered by Leichtenstern to be diagnostic of this form of invagination; for he states, in Zeimssen's *Cyclopædia of the Practice of Medicine*, vol. 7, p. 629, that "it can be almost always felt in colon and ileo-cæcal invaginations, but seldom in ileum invaginations." And further, according to Durham, 50 per cent. of cases are ileo-cæcal invaginations.

With regard to *treatment*, many cases have been recorded where success has followed the insufflation of air up the rectum, as well as the injection of warm water or of hot medicinal solutions, such as decoction of poppies, camomile infusion, &c. But so far as I am aware, the employment of *cold water* enemata (as was suggested in this case by Mr. Norton) has not been advocated before; but the success that has attended its use in this instance will certainly induce me to adopt it again in a similar case.

By constringing the swollen parts, subduing spasm, the cold water stimulates the muscular coat of the intestines to contract, and in this way, I believe, enables the stream of water to drive the invaginated parts before it.

* Read before the Bath and Bristol Branch of the British Medical Association.

Having no experience of *laparotomy*, I will only add that hitherto only 15 out of 58 recorded cases have recovered after this operation—which is a mortality of nearly 75 per cent. However, with a better recognition of this complaint, and an earlier adoption of operative interference, with proper antiseptic precautions, I doubt not this mortality will be considerably lessened in the future.

